# Comparisons of Motivation to Receive COVID-19 Vaccination and Related Factors between Frontline Physicians and Nurses and the Public in Taiwan: Applying the Extended Protection Motivation Theory

**DOI:** 10.3390/vaccines9050528

**Published:** 2021-05-20

**Authors:** Yen-Ju Lin, Cheng-Fang Yen, Yu-Ping Chang, Peng-Wei Wang

**Affiliations:** 1 Department of Psychiatry, School of Medicine, College of Medicine, Kaohsiung Medical University, Kaohsiung 80708, Taiwan; 1040457@kmuh.org.tw (Y.-J.L.); chfaye@cc.kmu.edu.tw (C.-F.Y.); 2 Department of Psychiatry, Kaohsiung Medical University Hospital, Kaohsiung 80708, Taiwan; 3 School of Nursing, The State University of New York, University at Buffalo, Buffalo, NY 14260, USA; yc73@buffalo.edu

**Keywords:** COVID-19, health care worker, motivation, prevention motivation theory, vaccination

## Abstract

This study aimed to compare the differences in motivation to receive a COVID-19 vaccination between frontline physicians and nurses and the Taiwanese public. The associations of threat and coping appraisals, as described in Protection Motivation Theory (PMT), with motivation to receive COVID-19 vaccination were compared between these groups, too. We recruited 279 frontline physicians and nurses and 768 members of the public by a Facebook advertisement. Participants’ motivation to receive COVID-19 vaccination, perceived severity of and vulnerability to COVID-19, self-efficacy and response efficacy of COVID-19 vaccination, response cost of COVID-19 vaccination, and knowledge about the mechanism of COVID-19 vaccination in light of PMT were determined. The results demonstrated that frontline health workers had higher motivation to receive COVID-19 vaccination than the public. Response efficacy and knowledge of COVID-19 vaccination were positively associated with motivation to receive COVID-19 vaccination in both frontline health workers and the public, whereas perceived vulnerability, perceived severity, and response cost of COVID-19 vaccination were positively associated with motivation in the public but not in frontline physicians and nurses. The factors related to motivation to receive COVID-19 vaccination should be considered when designing programs to increase motivation to receive COVID-19 vaccination among frontline health workers and the public.

## 1. Introduction

### 1.1. Frontline Health Care Workers in COVID-19 Pandemic

Frontline health care workers are a group at high risk of contracting COVID-19. A prospective cohort study showed that the incidence rate of COVID-19 infection in 99,795 frontline health care workers (HCWs) in the United Kingdom and United States was 3.96% from March to April 2020, a higher rate than that of the overall community [1]. Another study conducted in Qatar between 10 March and 24 June 2020 revealed that 10.6% of HCWs have tested positive for COVID-19 and that 11.6% of HCWs with a positive test were hospitalized [2]. A study using clinical data obtained from March 10 to 17 May 2020 reported an infection rate of 5.6% among HCWs and reported that 10.3% of infected HCWs transmitted the infection to their family and friends [3]. A review study investigating mortality among HCWs during the COVID-19 pandemic showed that one HCW died for every 100 HCWs infected and that the mortality rate of infected HCWs was higher for older HCWs than younger HCWs [4]. Many infected HCWs also struggle to return to their ordinary life after COVID-19 infection because they suffer from physical disability, low energy, cognitive symptoms, and affective symptoms [5,6]. Furthermore, some infected HCWs may develop suicidal ideation after a full recovery from COVID-19 [7].

With proactive containment efforts and comprehensive contact tracing, the number of COVID-19 cases in Taiwan has been low compared with other countries [8]. As of 18 April 2021, Taiwan had tested a total of 513,203 people and identified 1073 confirmed cases, nine of which were frontline HCWs, including two physicians and seven nurses [9]. Study data have indicated that the prevention of COVD-19 infection in frontline HCWs is an urgent issue.

### 1.2. Motivation to Have COVID-19 Vaccination among HCWs in Taiwan

Vaccination is a primary measure to stop the spreading of COVID-19 [10]. Taiwan has made substantial efforts to develop, procure, and distribute vaccines against COVID-19. The Ministry of Health and Welfare in Taiwan has formulated and announced the 2021 COVID-19 Vaccination Program [11] with the goal of reaching a nationwide vaccination rate of 65% to achieve herd immunity. Ten groups with different vaccination priorities have been defined. The first group, with the highest priority, is HCWs and is further divided into primary HCWs, who provide direct care to patients with suspected or confirmed cases of COVID-19, and secondary HCWs, who do not provide direct care to such patients [11]. A survey released by the Ministry of Health and Welfare in March 2021 revealed that 42.3% and 28.5% of primary and secondary HCWs, respectively, were willing to receive a COVID-19 vaccine [12]. Taiwan received 117,000 doses of the Oxford–AstraZeneca COVID-19 vaccine on 3 March 2021, and vaccination was made available to primary and secondary HCWs beginning 22 March [13]. The Oxford–AstraZeneca (Cambridge, UK) COVID-19 vaccine has been approved for an Emergency Use Listing by the World Health Organization [14]. The efficacy of the vaccine is 76.0% at preventing symptomatic COVID-19 beginning at 22 days following the first dose and 81.3% after the second dose [15]. Local pain, muscle ache, fatigue, and headache were the most common adverse reactions which occurred in Asian people [16]. Several cases of unusual thrombotic events in combination with thrombocytopenia were observed in patients after Oxford–AstraZeneca COVID-19 vaccination [17]. However, only 17,245 HCWs received Oxford–AstraZeneca COVID-19 vaccination over the first three weeks. The vaccination rates were 13.9% and 5.9% for primary and secondary HCWs, respectively [18]. HCWs are one of the priority groups for seasonal influenza vaccination in Taiwan; the coverage rate of seasonal influenza vaccination among HCWs in Taiwan ranged from 72.55% to 94.54% between 2004 and 2016 [19]. Compared with the rate of seasonal influenza vaccination, the rate of COVID-19 vaccination was significantly lower. The possible reasons accounting for the low COVID-19 vaccination rate in HCWs warrant further study.

### 1.3. Protection Motivation Theory

Examining and understanding the factors predicting the motivation to receive COVID-19 vaccination among frontline HCWs is critical for designing an effective intervention to increase their acceptance of vaccination [20]. Protection Motivation Theory (PMT) [21,22] has been used to explore the cognitive factors contributing to the acceptance of vaccination for respiratory infectious diseases (RIDs) such as influenza, measles, mumps, rubella, and pertussis [23,24,25]. According to PMT, threat appraisal and coping appraisal are two major cognitive processes that determine an individual’s motivation to adopt protective behaviors to reduce the risk of contracting RIDs [26]. Threat appraisal is based on the perceived severity of the health threat caused by RIDs and on the individual’s perceived vulnerability to RIDs. Coping appraisal is based on perceived self-efficacy (i.e., evaluating whether the individual can implement behaviors protecting against RIDs), response efficacy (i.e., evaluating whether these self-protective behaviors are effective in alleviating the health threats of RIDs), and response costs (e.g., money, time, and effort associated with engaging in self-protective behaviors to reduce the risk of RIDs) [24,26,27]. Perceived high severity, high vulnerability, high response efficacy, high self-efficacy, and low response costs contribute to high motivation to receive vaccination for influenza [26]. Moreover, research extended the PMT by including knowledge [28] and revealed that high knowledge about vaccine designs and protection mechanisms may increase an individual’s motivation to receive a vaccination [29]. Several studies have examined the predictors of HCW motivation to receive COVID-19 vaccination and revealed that low perceived risk of contracting COVID-19 [30], low trust in vaccine effectiveness [31,32], high concerns about unknown risks of the COVID-19 vaccines [31,33,34,35], and high concerns about time spent on vaccination [32] were associated with hesitancy to receive COVID-19 vaccination. Although the factors examined in these studies are included in the threat and coping appraisals of PMT, no study has examined the comprehensive PMT constructs that relate to frontline HCW motivation to receive COVID-19 vaccination. A study applying PMT found that threat appraisal but not coping appraisal predicted motivation to receive COVID-19 vaccination among university students in China [36]. Given that frontline HCWs face a direct threat from COVID-19, it is reasonable to hypothesize that frontline HCWs have a higher level of motivation to receive COVID-19 vaccination than members of the public do. However, as the COVID-19 pandemic is relatively mild in Taiwan, this hypothesis warrants examination. Moreover, frontline HCWs may have threat and coping appraisals toward COVID-19 that differ from those of the public [37]. Further study is required to understand whether threat and coping appraisals are differently associated with vaccination motivation in frontline HCWs and in the public. If differences exist between frontline HCWs and the public, programs to enhance motivation to receive COVID-19 vaccination should be specifically tailored accordingly.

### 1.4. Aims of This Study

The present study aimed to examine two research questions. First, is the level of motivation to receive COVID-19 vaccination in frontline HCWs different from that among the public? Second, are the associations of threat appraisal (i.e., perceived severity of and vulnerability to COVID-19) and coping appraisal (i.e., self-efficacy, response efficacy, and costs and knowledge of COVID-19 vaccination) with motivation to receive COVID-19 vaccination different between frontline HCWs and the public? Accordingly, the specific hypotheses are as follows:

**Hypothesis** **1** **(H1)**. *Frontline HCWs have higher motivation to receive COVID-19 vaccination than do members of the public.*

**Hypothesis** **2** **(H2)**. *The associations of threat and coping appraisals with motivation to receive COVID-19 vaccination are different between frontline HCWs and members of the public.*

## 2. Materials and Methods

### 2.1. Participants

Participants were recruited by using a Facebook advertisement on 15 October 2020 and 21 December 2020. Facebook (Facebook Inc., Menlo Park, CA, USA) users were eligible for this study if they were ≥20 years old and living in Taiwan. The Regulations on Human Trials in Taiwan stipulate that academic research recruiting minors younger than 20 years old shall obtain consent not only from the minors but also from their guardians. There were difficulties in obtaining consent from the guardians in online surveys; therefore, we only recruited individuals who were 20 or older into this study. The Facebook advertisement included a headline, main text, pop-up banner, and weblink to the research questionnaire website. We designed the advertisement to appear in the “News Feed” of Facebook, which is a streaming list of updates from the user’s connections (e.g., friends) and advertisers. We focused solely on News Feed advertisements (Facebook), as opposed to other Facebook advertising locations (e.g., the right column), because News Feed advertisements are more effective in terms of recruitment metrics for research studies. We targeted the advertisement to Facebook users by location (Taiwan) and by language (Mandarin Chinese), such that a given advertisement appeared on a user’s news feed as determined by a Facebook algorithm.

To ensure that frontline HCWs were recruited, we also posted the link of the Facebook advertisement to LINE (a direct messaging app, Tokyo, Japan) and to Facebook groups joined by HCWs. The headline and main text of the advertisement introduced the purpose and procedure of this study. Facebook users could press the button “Agree to participate” and go to the research questionnaire website or press the button “Disagree to participate” and leave the advertisement. In total, 1047 respondents pressed “Agree” to participate in this study, and 35 respondents pressed “Disagree” and did not participate. The Institutional Review Board of Kaohsiung Medical University Hospital approved this study (approval number: KMUHIRB-EXEMPT(I) 20,200,019 and date of approval: 13 October 2020). At the end of the online questionnaire, the participants were provided with web links to pages with information about COVID-19 on the websites of the Taiwan Centers for Disease Control, Kaohsiung Medical University Hospital, and the Medical College of National Cheng Kung University.

### 2.2. Measures

#### 2.2.1. Occupational Classification

We defined “frontline HCWs” as physicians and nurses responsible for providing direct care to patients with suspected or confirmed COVID-19. All other participants were classified as part of the public.

#### 2.2.2. Motivation to Receive COVID-19 Vaccination

The level of motivation to receive COVID-19 vaccination was assessed using one item: “Please rate your current willingness to receive a COVID-19 vaccine: 1 (very low) to 10 (very high)” [36] (Table 1).

#### 2.2.3. Constructs of PMT

The items for the Constructs of PMT are listed in Table 1. The constructs of PMT included perceived severity of COVID-19 (two items), perceived vulnerability to COVID-19 (four items), self-efficacy of receiving COVID-19 vaccination (one item), response efficacy of COVID-19 vaccination (six items), response cost of COVID-19 vaccination (three items), and knowledge about the mechanism of COVID-19 vaccination (three items). The items for measuring perceived severity and perceived vulnerability were adopted and transformed from the questionnaire developed by Liao et al. [38] for measuring risk perception of the H1N1 influenza to measure risk perception of COVID-19 [37]. The items for measuring self-efficacy, response efficacy, response cost, and knowledge were adopted from the Drivers of COVID-19 Vaccination Acceptance Scale (DrVac-COVID19S). The psychometrics of the DrVac-COVID19S has been examined in the previous study on Taiwanese and Mainland Chinese-speaking populations [39]. A higher score on each item indicated a higher level of threat appraisal, self-efficacy, response efficacy, response cost, and knowledge.

#### 2.2.4. Background Information Questions

Participants’ sex and age were collected.

### 2.3. Data Analysis

Data analysis was performed using SPSS 24.0 (SPSS Inc., Chicago, IL, USA). Sex and age were compared between frontline HCWs and the public by using the χ^2^ test and *t* test. Motivation to receive COVID-19 vaccination and PMT constructs related to COVID-19 vaccination were compared between frontline HCWs and the public by using analysis of covariance (ANCOVA) and controlling for the effects of sex and age. Due to the multiple comparisons, a two-tailed *p* value of <0.007 (0.05/7) indicated statistical significance.

The associations of occupational classification (frontline HCWs vs. the public) and of PMT constructs related to COVID-19 vaccination with motivation to receive COVID-19 vaccination were examined by using a multiple regression analysis. To examine the moderating effect of occupational classification, the interactions between occupational classification and the constructs of PMT were further investigated by using a multiple regression model to understand their associations with motivation to receive COVID-19 vaccination. A two-tailed *p* value of <0.05 indicated statistical significance.

## 3. Results

In total, 279 frontline HCWs and 768 members of the public participated in this study. The results of comparing sex, age, motivation to receive COVID-19 vaccination, and PMT constructs related to COVID-19 vaccination between frontline HCWs and the public are shown in Table 2. No sex difference was found between the two groups, whereas frontline HCWs were older than members of the public. ANCOVA results revealed that, controlling for the effects of sex and age, frontline HCWs had higher perceived vulnerability to COVID-19 and lower perceived response cost of COVID-19 vaccination than the public, whereas no differences between groups were found in the motivation to receive, self-efficacy of, response efficacy of, or knowledge of COVID-19 vaccination or in perceived COVID-19 severity.

Differences in motivation to receive COVID-19 vaccination between frontline HCWs and the public were further examined using multiple regression analysis (Model I in Table 3). The results demonstrated that after controlling for the effects of sex, age, and the PMT constructs related to COVID-19 vaccination, frontline HCWs had higher motivation to receive COVID-19 vaccination than did members of the public. The result supported H1.

The interactions between frontline HCWs and PMT constructs related to COVID-19 vaccination were further selected for use with the multiple regression model to examine their associations with motivation to receive COVID-19 vaccination (Model II). The results demonstrated that the perceived vulnerability, perceived severity, and response efficacy cost of COVID-19 vaccination among frontline HCWs were significantly associated with motivation to receive COVID-19 vaccination, indicating that frontline HCWs and members of the public had different associations between these factors and vaccination motivation. This result only partially supported H2.

The associations of PMT constructs related to the motivation to receive COVID-19 vaccination were further examined in frontline HCWs and in the public separately (Table 4). The results demonstrated that the positive associations of perceived vulnerability, perceived severity, and response cost of COVID-19 vaccination with motivation to receive COVID-19 vaccination existed among the public but not among frontline HCWs. Both response efficacy and knowledge of COVID-19 vaccination were positively associated with motivation to receive COVID-19 vaccination in both groups, whereas the association between self-efficacy and motivation to receive COVID-19 vaccination was not significant in either group.

## 4. Discussion

The results revealed that after controlling for the effects of sex, age, and the PMT constructs related to COVID-19 vaccination, frontline HCWs had higher motivation to receive COVID-19 vaccination than did the public. Both response efficacy and knowledge of COVID-19 vaccination were positively associated with motivation to receive COVID-19 vaccination in both groups, whereas perceived vulnerability, perceived severity, and response cost of COVID-19 vaccination were positively associated with motivation to receive COVID-19 vaccination among the public but not among frontline HCWs.

### 4.1. Motivation to Have COVID-19 Vaccination and PMT Related Factors in Frontline HCWs

The data supported the hypothesis that frontline HCWs have higher motivation to receive COVID-19 vaccination than do the public. The WHO declared that HCWs at high risk of exposure to COVID-19 should be prioritized for vaccination [10]. A high level of motivation to receive vaccination may increase vaccination rates. However, this study found that 34.1% of frontline HCWs rated their motivation to receive COVID-19 vaccination as 5 or below on a scale of 1 to 10, indicating that over one-third of frontline HCWs who are responsible for providing direct care to patients with suspected or confirmed COVID-19 feel hesitant to receive COVID-19 vaccination. Moreover, this study found no differences in self-efficacy, response efficacy, or knowledge of COVID-19 between the two groups. Although Taiwan has had a mild COVID-19 pandemic compared with other countries, frontline HCWs are still at high risk of contracting COVID-19. Vaccination hesitancy and low PMT coping appraisal of the COVID-19 vaccination may further increase the risk of HCWs contracting COVID-19. These results explain the low rate of vaccination among frontline HCWs since COVID-19 vaccination began in Taiwan. Whether similar results are found for frontline HCWs in other countries or areas is worth investigating.

### 4.2. PMT Factors Related to Motivation to Receive COVID-19 Vaccination in Frontline HCWs and in the Public

This study revealed various associations between PMT threat and coping appraisals with the motivation to receive COVID-19 vaccination for frontline HCWs and for the public. First, both response efficacy and knowledge of COVID-19 vaccination were positively associated with motivation to receive COVID-19 vaccination in both groups. In Taiwan, seasonal influenza mass vaccination campaigns are conducted annually. These campaigns both mitigate the effects of seasonal influenza and serve as functional exercises for mass vaccination operations during a pandemic [19]. It is possible that people in Taiwan may learn the effects of vaccination and gain knowledge about the reduced risk of RID contraction through vaccination. However, compared with the rate of seasonal influenza vaccination among HCWs in Taiwan [19], the rate of COVID-19 vaccination in the early stage was low. This indicates that experiences learned in the influenza pandemic may not be generalized to the COVID-19 pandemic.

Second, perceived vulnerability, perceived severity, and response cost of COVID-19 vaccination were positively associated with motivation to receive COVID-19 vaccination among the public but not among frontline HCWs. A study also found that perceived severity of COVID-19 was positively associated with motivation to receive COVID-19 vaccination among university students in China [36], indicating that increasing individuals’ threat appraisal of COVID-19 may be an effective method of increasing motivation to receive a COVID-19 vaccination for non-HCWs. Programs promoting COVID-19 vaccination for the public can use narrative communication as a tool (e.g., inviting the patients to convey the severity of contracting COVID-19) [40]. However, frontline HCWs may already be familiar with the severity of contracting COVID-19; therefore, increasing the threat appraisal of COVID-19 may not increase motivation to receive the COVID-19 vaccination. This study also demonstrated that the perceived cost of vaccination (including safety, possible side effects, money spent, and time spent) may have influences on the willingness of the public to receive COVID-19 vaccination. The results indicated that programs promoting COVID-19 vaccination for the public should mitigate uneasiness by providing clear information about the vaccination using language accessible to a general audience. Studies have revealed that high concern about the unknown risks of COVID-19 vaccines [31,33,34,35] and about time spent on vaccination [32] were primary reasons for vaccination hesitancy in HCWs and other employees in the health care system. However, the present study did not reveal the same result for frontline HCWs. Whether differences in the studied populations account for this discrepancy warrants further study.

Third, the present study did not reveal a significant association between self-efficacy and motivation for COVID-19 vaccination in either group. Self-efficacy of COVID-19 vaccination indicates an individual’s perception of their ability to receive COVID-19 vaccination. During the period of study, COVID-19 vaccines were unavailable in Taiwan, and participants may have had difficulty judging whether they could receive the COVID-19 vaccination.

### 4.3. Limitations

The present study had some limitations. First, this study has a limitation in terms of its representativeness. Recruiting participants using social media such as Facebook advertisement can deliver large numbers of participants quickly [41] and is a practical way to recruit participants during the pandemic. However, the use of social media to distribute invitations to participate in a survey may lead to problems of sample bias [42]. Access to Facebook is not yet universal; people are not equally motivated to use it. Facebook users may not be representative of the population. Moreover, online surveys cannot capture the responses of those who lack access to the internet, are nonliterate, or cannot proficiently use technology [43,44]. Facebook users may also consist of younger people among the general population [32]. Respondents may also share the survey with their friends and colleagues with similar interests or perspectives, which may lead to the over-representation of a particular viewpoint [45]. Second, the present study did not collect participants’ histories of physical health, which may influence their perception of COVID-19 and the necessity of vaccination. Third, the present study did not assess participants’ own and their families’ experiences of exposure to COVID-19, which may also influence their motivation to have COVID-19 vaccination. Fourth, there were 35 respondents who disagreed to participate in this study. We did not collect their characteristics or reasons why they disagreed to participate.

## 5. Conclusions

The present study found that response efficacy and knowledge of COVID-19 vaccination were positively associated with motivation to receive COVID-19 vaccination in both frontline HCWs and the public and could serve as the basis of programs for enhancing motivation to have COVID-19 vaccination regardless of the groups. It is recommended that government health departments invite medical professionals to introduce the efficacy and knowledge of COVID-19 vaccination in a convincing way to enhance the public’s appraisal of COVID-19 vaccination. Programs of enhancing frontline HCWs’ motivation to have COVID-19 vaccination may introduce the results of scientific studies examining the efficacy and knowledge of COVID-19 vaccination and the coverage rates of COVID-19 vaccination among frontline HCWs in other countries. The present study also found that some PMT factors such as perceived vulnerability, perceived severity, and response cost of COVID-19 vaccination were positively associated with motivation to receive COVID-19 vaccination among the public but not among frontline HCWs. It is recommended that government health departments develop group-specific programs for enhancing motivation to get vaccinated. For example, it is recommended that government health departments invite medical personnel to introduce COVID-19 threats to promote the public’s awareness; however, the introduction should not evoke a COVID-19 scare. Making COVID-19 vaccination easily available for the public is also important.

## Figures and Tables

**Table 1:** Motivation to Receive COVID-19 Vaccination and Constructs of the Extended PMT Related to COVID-19 Vaccination.

Measures	Items	Response Scale
*Motivation to receive COVID-19 vaccination*	Please rate your current willingness to receive a COVID-19 vaccine:	1 (very low) to 10 (very high)
*Perceived severity*	Item 1: Please rate the current level of your concern about COVID-19:	1 (very mild) to 10 (very severe)
	Item 2: How serious is COVID-19 relative to SARS?	1 (much less serious) to 5 (much more serious)
*Perceived vulnerability*	Item 1: How likely do you think it is that you will contract COVID-19 over the next month?	1 (never) to 7 (certain)
	Item 2: If you were to develop flu-like symptoms tomorrow, would you be:	1 (not at all worried) to 7 (extremely worried)
	Item 3: In the past week, have you ever worried about catching COVID-19?	1 (no, never think about it) to 5 (worried about it all the time)
	Item 4: What do you think your chances of getting COVID-19 over the next month are compared with others outside your family?	1 (not at all) to 7 (certain)
*Self-efficacy of COVID-19 vaccination*	I can choose whether to get a COVID-19 jab or not.	1 (strongly disagree) to 7 (strongly agree)
*Response efficacy of COVID-19 vaccination*	Item 1: Vaccination is a very effective way to protect me against COVID-19.	1 (strongly disagree) to 7 (strongly agree)
	Item 2: It is important that I get the COVID-19 shot.	
	Item 3: Vaccination greatly reduces my risk of catching COVID-19.	
	Item 4: The COVID-19 shot plays an important role in protecting my life and that of others.	
	Item 5: The COVID-19 shot will make an important contribution to my health and well-being.	
	Item 6: Getting the COVID-19 shot has a positive influence on my health.	
*Response cost of COVID-19 vaccination*	(1) Safety and possible side effects of vaccine, (2) cost of vaccine, and (3) time spent on vaccination will influence my willingness to get vaccinated for COVID-19.	1 (strongly disagree) to 4 (strongly agree)
*Knowledge about the mechanism of COVID-19 vaccination*	Item 1: I understand how the COVID-19 shot helps my body fight the COVID-19 virus.	1 (strongly disagree) to 7 (strongly agree)
	Item 2: I understand how vaccination protects me from COVID-19 well.	
	Item 3: How the COVID-19 jab works to protect my health is a mystery to me. *	

*: Reverse scoring.

**Table 2:** Comparisons of Sex, Age, Motivation to Receive COVID-19 Vaccination, and Constructs of PMT as Related to COVID-19 Vaccination Between Frontline Physicians and Nurses and the Public.

Variables	Frontline Physicians and Nurses (*N* = 279)	Public (*N* = 768)	χ^2^ or t or *F* ^b^	*p*
Sex, *n* (%)				
Female	161 (57.71)	456 (59.38)	0.236	0.627
Male	118 (42.29)	312 (40.62)		
Age, mean (*SD*; *range*)	37.73 (8.62; 22–58)	34.74 (9.82; 21–70)	4.501	<0.001
Motivation to receive COVID-19 vaccination, mean (*SD*) ^a^	6.75 (0.16)	6.53 (0.10)	1.432	0.232
Perceived vulnerability, mean (*SD*) ^a^	7.80 (0.20)	6.52 (0.12)	31.488	<0.001
Perceived severity, mean (*SD*) ^a^	6.32 (0.18)	6.80 (0.11)	5.133	0.024
Self-efficacy of COVID-19 vaccination, mean (*SD*) ^a^	5.06 (0.07)	4.96 (0.04)	1.523	0.217
Response efficacy of COVID-19 vaccination, mean (*SD*) ^a^	24.29 (0.42)	24.69 (0.25)	0.669	0.413
Response cost of COVID-19 vaccination, mean (*SD*) ^a^	5.42 (0.11)	5.91 (0.07)	13.754	<0.001
Knowledge of COVID-19 vaccination, mean (*SD*) ^a^	11.15 (0.24)	11.51 (0.14)	1.720	0.019

^a^: Estimated marginal mean after controlling sex and age by using analysis of covariance; ^b^: Analysis of covariance. PMT: Protection Motivation Theory.

**Table 3:** Factors Related to Motivation to Receive COVID-19 Vaccination: Multiple Regression Analysis.

Variables	Motivation to Have COVID-19 Vaccination					
	Model I			Model II		
	*B*	*SE*	*p*	*B*	*SE*	*p*
Frontline physicians and nurses ^a^	0.344	0.138	0.013	1.476	0.852	0.083
Male ^b^	0.154	0.121	0.205	0.142	0.120	0.238
Age	−0.005	0.006	0.388	−0.006	0.006	0.309
Perceived vulnerability	0.045	0.021	0.030	0.064	0.024	0.007
Perceived severity	0.047	0.023	0.039	0.082	0.026	0.002
Self-efficacy of COVID-19 vaccination	0.122	0.055	0.026	0.123	0.062	0.049
Response efficacy of COVID-19 vaccination	0.224	0.011	<0.001	0.211	0.012	<0.001
Response cost of COVID-19 vaccination	0.124	0.032	<0.001	0.170	0.036	<0.001
Knowledge of COVID-19 vaccination	0.066	0.018	<0.001	0.063	0.020	0.002
Frontline physicians and nurses x Perceived vulnerability				−0.099	0.048	0.039
Frontline physicians and nurses x Perceived severity				−0.130	0.050	0.009
Frontline physicians and nurses x Self-efficacy to have COVID-19 vaccination				0.032	0.125	0.798
Frontline physicians and nurses x Response efficacy of COVID-19 vaccination				0.044	0.027	0.103
Frontline physicians and nurses x Response cost of COVID-19 vaccination				−0.164	0.071	0.021
Frontline physicians and nurses x Knowledge of COVID-19 vaccination				0.015	0.045	0.745
Adjusted *R*^2^	0.477			0.493		

^a^: Public as the reference; ^b^: Female as the reference.

**Table 4:** Constructs of PMT as Related to Motivation to Receive COVID-19 Vaccination in Frontline Physicians and Nurses and the Public: Multiple Regression Analysis.

Variables	Motivation to Have COVID-19 Vaccination					
	Frontline Physicians and Nurses			Public		
	*B*	*SE*	*p*	*B*	*SE*	*p*
Male ^a^	0.137	0.220	0.534	0.138	0.143	0.334
Age	0.005	0.012	0.711	−0.009	0.007	0.196
Perceived vulnerability	−0.035	0.039	0.364	0.064	0.024	0.008
Perceived severity	−0.044	0.040	0.267	0.082	0.027	0.002
Self-efficacy of COVID-19 vaccination	0.157	0.101	0.120	0.120	0.064	0.062
Response efficacy of COVID-19 vaccination	0.252	0.022	<0.001	0.211	0.012	<0.001
Response cost of COVID-19 vaccination	−0.001	0.058	0.985	0.170	0.037	<0.001
Knowledge of COVID-19 vaccination	0.080	0.037	0.033	0.062	0.020	0.002
Adjusted *R*^2^	0.581			0.459		

^a^: Female as the reference. PMT: Protection Motivation Theory.

## Data Availability

The data will be available upon reasonable request to the corresponding authors.
